# ZiYinHuaTan Recipe Inhibits Cell Proliferation and Promotes Apoptosis in Gastric Cancer by Suppressing PI3K/AKT Pathway

**DOI:** 10.1155/2020/2018162

**Published:** 2020-04-22

**Authors:** Jiahui Yu, Shangjin Song, Jianpeng Jiao, Xuan Liu, Huirong Zhu, Lijuan Xiu, Dazhi Sun, Qi Li, Xiaoqiang Yue

**Affiliations:** ^1^Department of Traditional Chinese Medicine, Changzheng Hospital, Naval Medical University, Shanghai 200003, China; ^2^School of Traditional Chinese Medicine, Naval Medical University, Shanghai 200433, China; ^3^Cancer Institute, Shuguang Hospital, Shanghai University of Traditional Chinese Medicine, Shanghai 201203, China

## Abstract

In recent years, traditional Chinese medicine has played an important role in the treatment of gastric cancer in China. ZiYinHuaTan (ZYHT) recipe was developed for advanced gastric cancer and had shown its promising value in the clinic. In this study, we explore the effect of ZYHT on gastric cancer in vitro and in vivo. ZYHT can inhibit tumor growth and improve the general condition of mice in subcutaneous transplantation nude mice models of gastric cancer. And ZYHT can also inhibit cell proliferation and blocked the cells in G0/G1 to induce cell apoptosis in HGC27 and MGC803 cells. Then, network pharmacology analysis showed that ZYHT may exert antitumor effect mainly through PI3K/AKT signaling pathway. Furthermore, the expression of PI3K, p-Akt, CyclinD1, and Bcl-2 was detected in vitro and in vivo. The results showed that ZYHT could decrease the expression of PI3K, CyclinD1, and Bcl-2 both in vitro and in vivo. These results suggested that ZYHT could be used as a method for the treatment of developed gastric cancer.

## 1. Introduction

Gastric cancer (GC) is the third leading cause of cancer-related mortality in the world and the second in China [1, 2]. Surgery is currently the curative treatment for gastric cancer; chemotherapy, radiotherapy, and even gene therapy are considered the main adjuvant therapy methods. However, accounting for the late detection, drug resistance, and toxic side effects, new therapies should be developed to improve the prognosis of GC. In recent years, traditional Chinese medicine (TCM) has played an important role in the treatment of gastric cancer in China. Various studies have shown that it is effective in preventing gastric cancer, reducing recurrence, alleviating pain, improving quality of life, and prolonging survival.

ZiYinHuaTan (ZYHT) recipe, developed for the advanced gastric cancer, was based on the hypothesis of “tumor-phlegm microenvironment” and had shown its promising value in the clinic [3–5]. ZYHT is composed of *Lily*, *Pinellia*, and *Hedyotis diffusa*. Several components of ZYHT have recently been reported to exert antitumor effects in several cancers. However, the potential role of ZYHT in the treatment of GC and the precise mechanisms have not yet been clearly addressed.

Network pharmacology, as a novel strategy to identify the active compounds of herbs and their therapeutic targets, was first proposed by Professor Hopkins [6] and has increasingly become a tool to systematically reveal the function of a complex biological system [7, 8], which coincides with the overall concept of TCM and provides a new approach to explore the pharmacological mechanism of TCMs [9, 10].

**Figure 1:**
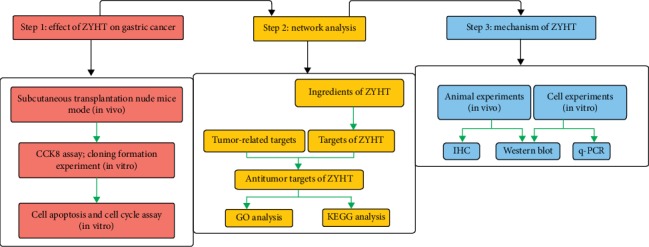
Flowchart of the systems pharmacology approach for uncovering the pharmacological mechanisms of ZiYinHuaTan (ZYHT) recipe actions on gastric cancer by integrating target identification, network analysis, and experimental validation. (a) Effect of ZYHT on the growth of gastric cancer. (b) Network analysis to explore the possible targets and mechanisms of ZYHT for gastric cancer. (c) Experimental validation in vivo and in vitro to verify the mechanism of action of ZYHT on gastric cancer.

In the present study, in vitro and in vivo studies were performed to evaluate the role of ZYHT on gastric cancer. Furthermore, we employed a system pharmacology approach to investigate the active components, potential targets, and therapeutic mechanisms of ZYHT and we detected the mechanisms by which ZYHT inhibits inhibiting cell proliferation and promotes apoptosis in gastric cancer through PI3K/AKT pathway (Figure 1).

## 2. Materials and Methods

### 2.1. Preparation of the Extracts for ZYHT

All decoction components (*Lily*, *Pinellia*, and *Hedyotis diffusa*) were purchased from Shanghai Lei Yun Shang Pharmacy Co., Ltd. (China). The extracts of ZYHT were extracted by Shanghai Changzheng Hospital Manufacturing Laboratory (Shanghai, China). The extracts of ZYHT were dissolved at a concentration of 10 mg/mL as a stock solution in RPMI-1640 medium containing 10% fetal bovine serum (FBS), followed by ultrasonic mixing overnight and filtration with a 0.22 *μ*m filter.

### 2.2. Cell Culture

The human gastric cancer cell lines HGC27 and MGC803 were purchased from the Cell Resource Center of Shanghai Institutes for Biological Sciences (Shanghai, China). Cells were maintained in RPMI-1640 containing 10% FBS, penicillin (100 U/mL), and streptomycin (100 mg/mL) (Invitrogen, Carlsbad, CA, USA) at 37°C in a 5% CO_2_ atmosphere.

### 2.3. CCK-8 Assay

Cell proliferation assay was conducted using the cell count kit CCK-8 (Sigma). Briefly, cells were seeded in 96-well plates at 1 × 10^4^ cells/well. When the cells reached 60% confluence, the medium was removed and replaced with fresh medium containing varying concentrations of ZYHT and incubated for 48 h. The absorbance was read at 450 nm using a microplate enzyme-linked immunosorbent assay reader (Labsystems Dragon, Wellscan). All experiments were performed with 5 wells per experiment and repeated at least three times. IC10, IC20, and IC50 (the half-maximal inhibitory concentration) were estimated according to the following formula, respectively: IC_50_ = lg − 1 [Xm − *i*(ΣP − 0.5)].

### 2.4. Cloning Formation Experiment

HGC27 and MGC803 cells in the logarithmic growth phase were uniformly inoculated into 6-well plates at 500 cells/well and cultured in a 37°C, 5% CO_2_ incubator. After the cells were attached, the experimental group was added with different concentrations of ZYHT. When macroscopically visible clones were formed, the culture was terminated, then washed 3 times with PBS, fixed with methanol for 20 min, and stained with Giemsa for 30 min. After drying, photographs were taken and the number of clones was counted, and the experiment was repeated 3 times.

### 2.5. Cell Apoptosis and Cell Cycle Assay

Flow cytometry (FCM) was performed for cell apoptosis assay. Cells were seeded into 6-well plates at a density of 1 × 10^5^ cells/well. Then, cells were resuspended in Annexin-binding buffer, and 5 *μ*L Annexin V-FITC and 1 *μ*L PI were appended. Afterwards, 5 *μ*L Annexin V-FITC and 1 *μ*L PI double staining (BD Biosciences, Franklin 177 Lakes, NJ, USA) were used. The apoptotic cells were performed using flow cytometric analyses (FACSCalibur, BD Biosciences) and the Flowjo software (Tree Star Corp, Ashland, USA) was used to analyze the results. For cell cycle analysis, HGC27 and MGC803 cells were fixed with 70% ethanol overnight and stained with PI (0.1 mg/mL) in the presence of Ribonuclease A (Takara) for 30 min at room temperature. The cell cycle distribution was analyzed by flow cytometry (FACSCalibur).

### 2.6. Animal Experiments

200 *μ*L single-cell suspensions of HGC27 cells (2 × 10^6^) were injected into the subcutaneous area of male BALB/c nude mice (4–6 weeks old) obtained from Sino-British SIPPR/BKLab Animal Co., Ltd. (Shanghai, China). When the transplanted tumors reached 100 mm^3^, the mice were randomized into six groups of 6 animals each. ZYHTs with low dose (940 mg/kg/day), middle dose (1880 mg/kg/day), and high dose (3760 mg/kg/day) and Xeloda (267 mg/kg/day) with control physiological saline were intragastrically administered every day for the above six groups. The body weight of the animals and the two perpendicular diameters (A and B) of the tumor were recorded every 3 days. And tumor volume (*V*) was estimated according to the following formula: *V* = *π*/6*x*[(*A* + *B*)/2]3. The curve of the tumor growth was drawn according to tumor volume and time of transplantation.

### 2.7. Network Pharmacology Analysis

The main components of ZYHT were screened from TCMSP (http://ibts.hkbu.edu.hk/LSP/tcmsp.php) [11], HIT (http://lifecenter.sgst.cn/hit) [12], TCMID (http://www.megabionet.org/tcmid) [13],and TCM Database@Taiwan (http://tcm.cmu.edu.tw/) [14] which satisfy both OB ≥ 30% and DL ≥ 0.18 that were selected as effective active components. We obtained the protein targets of effective active components through the DRAR-CPI server and collected the tumor-associated targets through DrugBank [15] (https://www.drugbank.ca/) and Therapeutic Targets Database (http://bidd.nus.edu.sg/BIDD-Databases/TTD/TTD.asp) [16] and then matched them. We constructed the component-target network by Cytoscape 3.5.1 (http://www.cytoscape.org/) [17]. We performed gene ontology (GO) biological process and KEGG pathway analysis of antitumor targets of ZYHT using DAVID (https://david.ncifcrf.gov/) [18].

### 2.8. Western Blot

HGC27 and MGC803 cells (2 × 10^5^) were seeded in 6-well plates and cultured for 48 h with various interventions. The cells were subsequently collected and decomposed by 100 *μ*L loading buffer. Proteins in the total cell lysate were separated by SDS-PAGE (10% separation gel and 5% spacer gel) and transferred to PVDF membranes. Membranes were placed in blocking solution for 2 h. Next, the membranes were incubated with the primary antibodies and subsequently the HRP-conjugated secondary antibodies. All experiments were repeated three times.

### 2.9. RT-qPCR

Real-time PCR was performed in Applied Biosystems 7300 System (Applied Biosystems Deutschland GmbH), according to the instructions of the Premix Ex Taq Kit (Takara, Dalian, China). The primers were designed as follows: GAPDH, 5-GGTGGTCTCCTCTGACTTCAA-3 (forward) and 5-CCAAATTCGTTGTCATACCAG-3 (reverse); PI3K, AKT, CCD1, BCL-2, BAX, and P53. All assays were repeated three times.

### 2.10. Immunohistochemistry

Expressions of PI3K, p-AKT, Cyclin D1, and BCL-2 in specimens were determined by the protocol of immunohistochemistry (IHC) described previously [19]. We used antibodies against PI3K (1 : 200, CST, USA), p-AKT (1 : 200, CST, USA), CyclinD1 (1 : 200, CST, USA), and BCL-2 (1 : 200, CST, USA). Sections were observed under the microscope.

### 2.11. Statistical Analysis

Statistical analysis was performed using SPSS 21.0. All data are expressed as the mean ± SD of at least three independent experiments. One-way analysis of variance (ANOVA) was used to analyze the difference between the control group and the ZYHT group. A difference was considered statistically when the corresponding *P* < 0.05.

## 3. Results

### 3.1. ZYHT Inhibits Tumor Growth in Subcutaneous Transplantation Nude Mice Models

**Figure 2:**
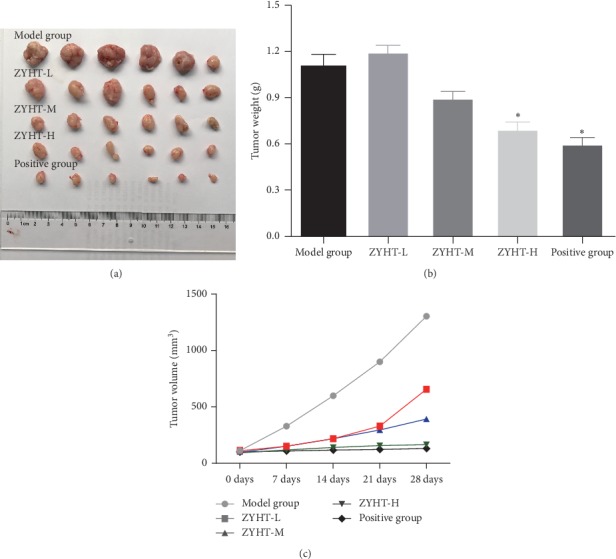
Effect of ZYHT on tumor growth of nude mice in 28 days. (a) Effect of ZYHT on tumor growth of subcutaneously transplanted tumors in mice (*n* = 6). (b) Effect of ZYHT on tumor weight of nude mice with subcutaneous tumor model of gastric cancer (*n* = 6). (c) Effect of ZYHT on tumor volume of nude mice with subcutaneous tumor model of gastric cancer (*n* = 6). ZYHT-L: ZiYinHuaTan aqueous extract low group, 0.94 g/kg; ZYHT-M: ZiYinHuaTan aqueous extract middle group, 1.88 g/kg; ZYHT-H: ZiYinHuaTan aqueous extract high group, 3.76 g/kg. *P* < 0.05^*∗*^ vs control group.

To detect the effect of ZYHT in tumor growth in vivo, we used a subcutaneous transplantation nude mice model of HGC-27 cells. Compared with blank control, ZYHT therapy significantly slowed down the growth rate of tumors and reduced the tumor volume (*P* < 0.0) (Figure 2).

Otherwise, ZYHT can improve the general condition of subcutaneously transplanted tumor model nude mice. One week after modeling, the normal control mice responded flexibly, with a ruddy color, an active activity, and active feeding. The other 5 groups of mice showed impotence, laziness in activity, and loose and dull skin. The drug was administered on the 15th day after modeling, and the mice in the normal group had no special performance. The other 5 groups of mice were wilting, the skin was dull, and the food was not actively taken.

### 3.2. ZYHT Inhibits Proliferation of Gastric Cancer Cells

**Figure 3:**
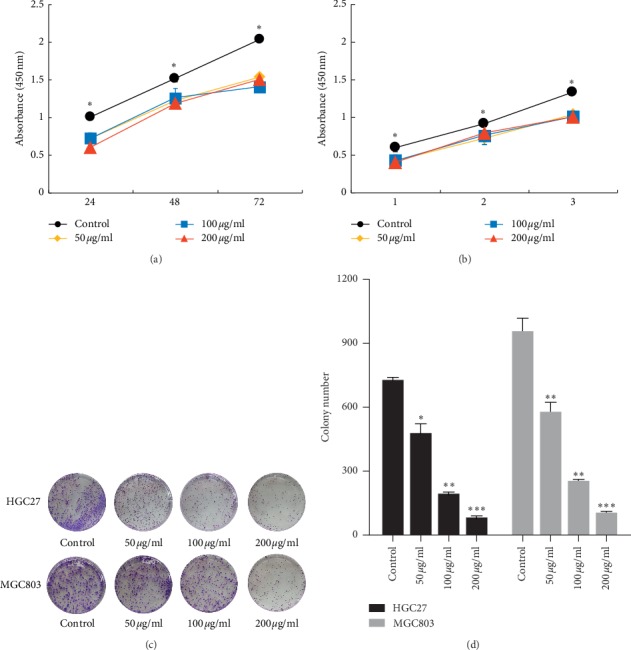
Effect of ZYHT on the proliferation of gastric cancer cells (*n* = 3). (a) Changes in the proliferation level of HGC27 cells after the intervention of ZYHT. (b) Changes in the proliferation level of MGC803 cells after the intervention of ZYHT. (c) Comparison of the number of clones formed in each group after the intervention of ZYHT. ^*∗*^*P* < 0.05 vs control group, ^*∗∗*^*P* < 0.01 vs control group, and ^*∗∗∗*^*P* < 0.001 vs control group.

To investigate the growth inhibitory effect of ZYHT on GC progression in vitro, HGC-27 and MGC-803GC cells were treated with ZYHT. We evaluated cell proliferation using the CCK-8 assay. Our data revealed that the doses of IC10, IC25, and IC50 were 52.24 *μ*g/mL, 104 *μ*g/mL, and 226.68 *μ*g/mL. And then, the concentration of the low, medium, and high doses of ZYHT was determined to be 50, 100, and 200 *μ*g/mL. As shown in Figures 3(a) and 3(b), ZYHT significantly decreased the viability of HGC-27 and MGC-803 cells in a time-dependent manner. The colony formation experiment showed that in each ZYHT group (low, medium, or high dose) the cell proliferation rate is lower than the blank group (Figure 3(c)). It is indicated that different doses of ZYHT inhibited the proliferation of gastric cancer cells.

### 3.3. ZYHT Blocks the GC Cell Cycle Arrest at the G0/G1 Checkpoint and Induces Cell Apoptosis

**Figure 4:**
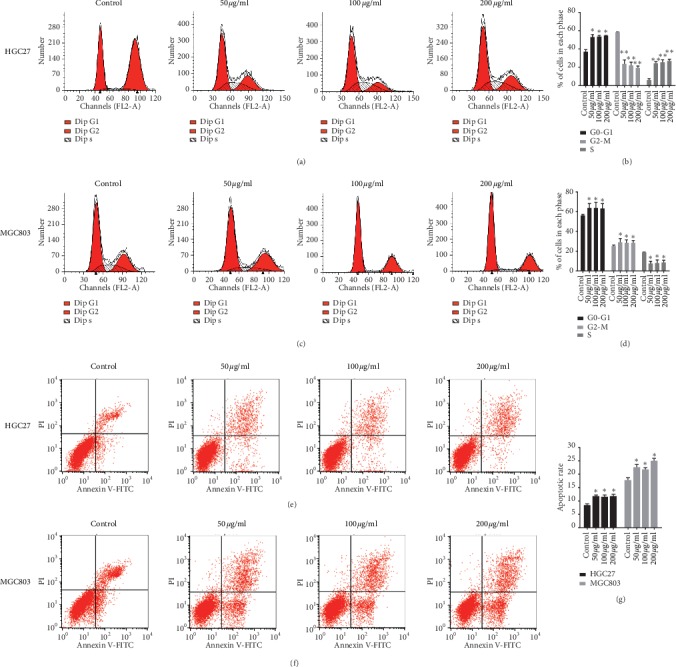
Effect of ZYHT on cell cycle and apoptosis of gastric cancer cells (*n* = 3). (a) Effect of ZYHT on cell cycle of HGC27. (b) Effect of ZYHT on cell cycle of HGC27. (c) Effect of ZYHT on cell cycle of MGC803. (d) Effect of ZYHT on cell cycle of MGC803. (e) Comparison of the apoptosis rate of HGC27 cells after the intervention of ZYHT. (f) Comparison of the apoptosis rate of MGC803 cells after the intervention of ZYHT. (g) Comparison of apoptosis rate of HGC27 and MGC803 cells after the intervention of ZYHT. ^*∗*^*P* < 0.05 vs control group, ^*∗∗*^*P* < 0.01 vs control group, and ^*∗∗∗*^*P* < 0.001 vs control group.

To explore the mechanisms of the antiproliferative effect of ZYHT, flow cytometry (FCM) was used to detect the effect of ZYHT on the cell cycle of gastric cancer. As shown in Figures 4(a)–4(d), the number of cells in the G1 phase increased and in the S phase decreased significantly compared with the control group (*P* < 0.05) after treated with ZYHT in HGC27 and MGC803 cells. And the proliferation index (PI) of the ZYHT group was significantly lower than that of the control group (*P* < 0.05).

FCM analysis of Annexin V/PI double staining showed that the apoptosis rates of HGC27 cells induced by 0, 50, 100, and 200 *μ*g/mL of ZYHT were 12.91 ± 0.52, 16.80 ± 0.45, 17.76 ± 0.48, and 17.92 ± 0.28%, respectively (Figure 4(e)). The apoptotic rates of MGC803 cells were 17.86 ± 0.46, 22.74 ± 0.53, 24.59 ± 0.45, and 25.63 ± 0.31%, respectively (Figure 4(f)). The apoptosis rate of HGC27 cells and MGC803 cells treated with low-dose, medium-dose, and high-dose interventions of ZYHT was significantly increased compared with the blank group (*P* < 0.05) (Figure 4(g)). These data suggested that ZYHT induces apoptosis of gastric cancer cells.

### 3.4. Network Pharmacology Analysis of ZYHT

By November 2017, a total of 284 kinds of chemical components (supplementary (available here)) in ZYHT were collected. There are 25 effective chemical components satisfying OB ≥ 30% and DL ≥ 0.18 obtained. Through the molecular docking of the DRAR-CPI server, 294 potential gene targets (supplementary) were obtained corresponding to 25 chemical components. And a total of 833 tumor-related targets were obtained by searching the DrugBank and TTD databases. Then, after matching with the 294 potential targets of ZYHT, a total of 92 antitumor targets were identified.

**Figure 5:**
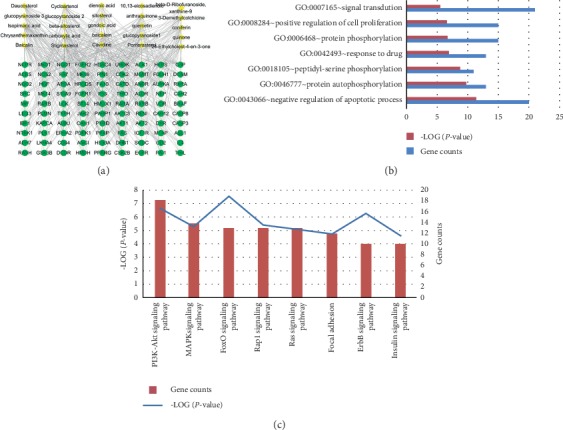
Flowchart of the approach for uncovering the pharmacological mechanisms of ZiYinHuaTan (ZYHT) recipe on gastric cancer (GC) by network analysis and experimental validation. (a) Experimental validation in vivo and in vitro to evaluate the role of ZYHT on GC. (b) Network analysis of ZYHT to explore the pharmacological mechanisms of ZYHT on GC. (c) Experimental validation to decipher the mechanisms of ZYHT on GC.

The component-target network diagram was formed by analyzing 25 effective chemical components of ZYHT and their corresponding antitumor-related targets through Cytoscape (Figure 5(a)). Gene ontology (GO) analysis on 92 potential antitumor targets (supplementary) shows that ZYHT may mainly regulate biological processes such as phosphorylation, apoptosis, and cell proliferation in cells (Figure 5(b)). The KEGG pathway analysis found that ZYHT may exert antitumor effect mainly through PI3K/AKT signaling pathway (Figure 5(c)).

### 3.5. Effect of ZYHT on PI3K/Akt Signaling Pathway In Vitro

**Figure 6:**
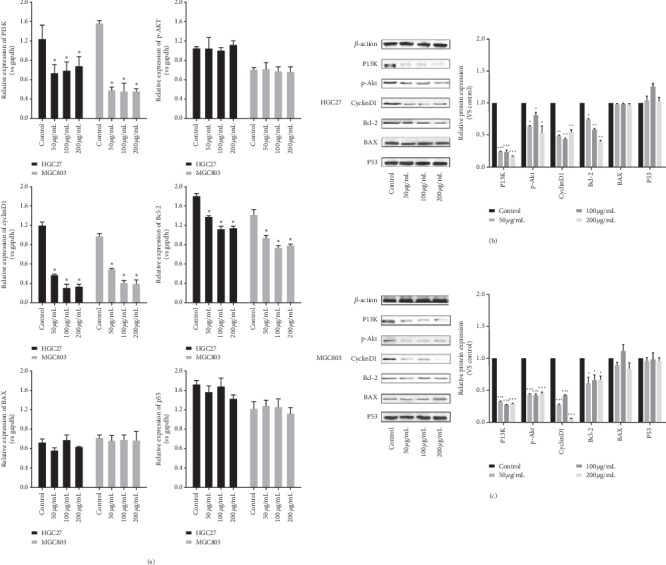
Effect of ZYHT on mRNA and protein expression of gastric cancer cells (*n* = 3). (a) Effect of ZYHT on RNA expression of HGC27 and MGC803. (b) Effect of ZYHT on the protein expression of HGC27. (c) Effect of ZYHT on the protein expression of MGC803. ^*∗*^*P* < 0.05 vs control group, ^*∗∗*^*P* < 0.01 vs control group, and ^*∗∗∗*^*P* < 0.001 vs control group.

To investigate whether ZYHT inhibits GC cell proliferation and promotes apoptosis through the PI3K/Akt signaling pathway, we performed q-PCR and WB experiments to verify the effect of ZYHT on PI3K/Akt signaling pathway. Q-PCR experiments showed that ZYHT could decrease the mRNA expression of PI3K, CyclinD1, and Bcl-2 in MGC803and HGC27 significantly, while there was no significant effect on the expression of AKT, BAX, and p53 (Figure 6(a)). As shown in Figures 6(b) and 6(c), compared with normal HGC27 and MGC803 cells, the BAX and P53 protein levels treated with ZYHT did not change significantly, while the protein expression of PI3K, p-Akt, CyclinD1, and Bcl-2 was significantly inhibited (*P* < 0.05).

### 3.6. Effect of ZYHT on PI3K/Akt Signaling Pathways In Vivo

**Figure 7:**
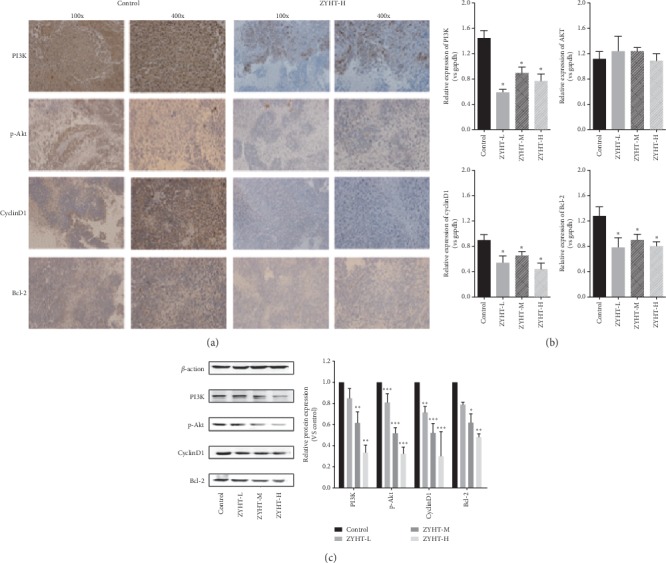
Effect of ZYHT on RNA and protein expression of gastric cancer (*n* = 3). (a) Effect of ZYHT on protein expression of gastric cancer (*n* = 3). (b) Effect of ZYHT on RNA expression of gastric cancer (*n* = 3). (c) Effect of ZYHT on protein expression of gastric cancer (*n* = 3). ZYHT-L: ZiYinHuaTan aqueous extract low group, 0.94 g/kg; ZYHT-M: ZiYinHuaTan aqueous extract middle group, 1.88 g/kg; ZAuE3: ZiYinHuaTan aqueous extract high group, 3.76 g/kg. ^*∗*^*P* < 0.05 vs control group, ^*∗∗*^*P* < 0.01 vs control group, and ^*∗∗∗*^*P* < 0.001 vs control group.

In the in vivo experiment, the data showed that ZYHT can downregulate the protein expression of PI3K, AKT, CyclinD1, and Bcl-2 in the subcutaneous xenograft GC tumor tissues (Figures 7(a) and 7(c)). The results of q-PCR showed that the mRNA expressions of PI3K, CyclinD1, and Bcl-2 were decreased significantly compared with the control group (Figure 7(b)) (*P* < 0.05), but they have no effect on the mRNA expression of AKT. These findings suggested that ZYHT might inhibit proliferation and promotes apoptosis through the PI3K/Akt signaling pathway in GC.

## 4. Discussion

The low early diagnosis rate of GC and the limited treatment options for advanced GC make it one of the most deadly of cancers [20]. Currently, chemotherapy, surgery, and radiation therapy are the major treatment options for GC. Since chemotherapeutics have the insurmountable problems of multidrug resistance and adverse effects (such as fatigue, anemia, vomiting, decreased neutrophils, thrombocytopenia, diarrhea, and nausea), and resection or radical surgery can cause surgical trauma, malnutrition, and other complications, the quality of life and 5-year overall survival rate for GC patients remain low. In China, Chinese herbal medicines, due to clear curative effects and low toxicity, are increasingly used as alternative and complementary approaches for the prevention and treatment of gastric cancer [21, 22].

Published studies have shown that adjunctive TCM therapies display remarkable advantages, including improving life quality, ameliorating symptoms, maintaining organ functions, reducing side effects of chemotherapy or radiotherapy, and extending survival time in patients with advanced gastric cancer. In our previous study, we found that phlegm-eliminating herbs, such as *Pinellia*, have significant anticancer effects [23–27]. Based on such clinical findings, we aimed to explore the anticancer effect of ZYHT in vitro and in vivo and investigated the underlying mechanism. ZYHT significantly inhibited proliferation and induced apoptosis in HGC27 and MGC803 cells and inhibited tumor growth and improved the general condition of mice in subcutaneous transplantation nude mice models.

To explore the molecular mechanism involved in ZYHT-induced anticancer activity, we employed network pharmacology analysis to determine the components, targets, and underlying mechanism of ZYHT. Network pharmacology abandons the traditional thinking of “one disease, one target, one drug” and constructs a “multidrugs and multitargets” network structure to achieve the goal of predicting drug targets as a whole [28].

In this study, network analysis revealed a total of 284 components and 294 gene targets in ZYHT. And 25 effective components and their 92 corresponding antitumor targets were analyzed. GO analysis shows that ZYHT may mainly regulate biological processes such as phosphorylation, apoptosis, and cell proliferation. And the KEGG pathway analysis found that ZYHT may exert an antitumor effect through PI3K/AKT, MAPK, FoxO, Rap1, Ras, and ErbB signaling pathway. These results suggested that ZYHT-anticancer efficacy was through the synergistic effect of multicompounds, multitargets, and multipathways.

Among those numerous signaling pathways, the PI3K/AKT pathway is one of the most critical. In the present study, we demonstrated that ZYHT can modulate PI3K/Akt signaling pathway by inhibiting PI3K expression in vitro and in vivo. As crucial coordinators of the intracellular signaling pathway, PI3K/Akt pathway could regulate multiple signaling pathways, including cell proliferation, apoptosis, mobility, and metabolism in gastric carcinogenesis and progression [29, 30]. PI3Ks are the second messengers produced by phosphoinositide phosphorylate at the D-3 position of the inositol ring. AKT, a downstream effect of PI3K, can regulate several biological properties in GC, including cell growth, survival, and apoptosis [31]. In this study, it was found that PI3K and P-AKT, the activated form of AKT, are downregulated following the treatment with ZYHT.

Bcl-2 is the most apoptotic effector of Bcl-2 families, which are important regulators in the PI3K/AKT pathway [32]. Cyclin D1, the downstream regulator of the PI3K/AKT pathway, is an important protein related to the G0/G1 cell cycle checkpoint [33]. The increased expression of Cyclin D1 contributes significantly to tumor development and malignant transformation [34, 35]. The present results showed that ZYHT could decrease the expression of Bcl-2 and Cyclin D1, which may be the main mechanism of ZYHT promoting apoptosis and inhibiting proliferation on gastric cancer cells.

## 5. Conclusions

In summary, our results discovered that ZYHT can inhibit cell proliferation and promote apoptosis in gastric cancer, and ZYHT-mediated PI3K/Akt signal pathway may account for its potential mechanism for proliferation and apoptosis. These results suggested that ZYHT could be used as a method for the treatment of developed gastric cancer.

## Data Availability

The data used to support the findings of this study are available from the corresponding author upon request.

## Supplementary Materials

Supplementary Materials The supplementary contains chemical components and potential targets of ZYHT and tumor-related targets. 284 kinds of chemical components (sheet 1) in ZYHT were collected from TCMSP, HIT, TCMID, and TCM Database@Taiwan. 294 potential targets (sheet 2) of ZY HT were obtained through the DRAR CPI server, which were demonstrated using UniProt ID. 833 tumor-related targets (sheet 3) were obtained by searching the DrugBank and TTD databases, which were also demonstrated using UniProt ID.
